# Reliability and validity of the Spanish version of the Child Health and Illness Profile (CHIP) Child-Edition, Parent Report Form (CHIP-CE/PRF)

**DOI:** 10.1186/1477-7525-8-78

**Published:** 2010-08-02

**Authors:** Maria-Dolors Estrada, Luis Rajmil, Vicky Serra-Sutton, Cristian Tebé, Jordi Alonso, Michael Herdman, Anne W Riley, Christopher B Forrest, Barbara Starfield

**Affiliations:** 1 Agència d'Avaluació de Tecnologia i Recerca Mèdiques, Roc Boronat 81-95 2nd Floor Barcelona 08005, Spain; 2 CIBER de Epidemiología y Salud Pública CIBERESP, Dr Aiguader 88, Barcelona 08003, Spain; 3 Institut Municipal d'Investigació Mèdica (IMIM-Hospital del Mar), Dr Aiguader 88, Barcelona 08003, Spain; 4 Johns Hopkins School of Public Health, 2008 South Road Baltimore, Maryland, USA; 5 Children's Hospital of Philadelphia, Adolescent Medicine Department, 3535 Market Street - Suite 1371, Philadelphia, PA 19104, USA

## Abstract

**Background:**

The objectives of the study were to assess the reliability, and the content, construct, and convergent validity of the Spanish version of the CHIP-CE/PRF, to analyze parent-child agreement, and compare the results with those of the original U.S. version.

**Methods:**

Parents from a representative sample of children aged 6-12 years were selected from 9 primary schools in Barcelona. Test-retest reliability was assessed in a convenience subsample of parents from 2 schools. Parents completed the Spanish version of the CHIP-CE/PRF. The Achenbach Child Behavioural Checklist (CBCL) was administered to a convenience subsample.

**Results:**

The overall response rate was 67% (n = 871). There was no floor effect. A ceiling effect was found in 4 subdomains. Reliability was acceptable at the domain level (internal consistency = 0.68-0.86; test-retest intraclass correlation coefficients = 0.69-0.85). Younger girls had better scores on Satisfaction and Achievement than older girls. Comfort domain score was lower (worse) in children with a probable mental health problem, with high effect size (ES = 1.45). The level of parent-child agreement was low (0.22-0.37).

**Conclusions:**

The results of this study suggest that the parent version of the Spanish CHIP-CE has acceptable psychometric properties although further research is needed to check reliability at sub-domain level. The CHIP-CE parent report form provides a comprehensive, psychometrically sound measure of health for Spanish children 6 to 12 years old. It can be a complementary perspective to the self-reported measure or an alternative when the child is unable to complete the questionnaire. In general, the results are similar to the original U.S. version.

## Background

Patient reported outcome measures (PRO) such as perceived health status or health-related quality of life (HRQOL) are primarily based on self-reported information. Until recently, PRO assessment in children has relied on parent-proxy reporting. Over the past several years, a number of self-reported instruments have been developed for school-aged children [1], and this has prompted the question of whether self-report, parent-report, or both perspectives on PRO should be collected. Despite the increasing number of studies considering health status and HRQOL in children, information on the factors that contribute to parent-child agreement levels remains limited [2]. Agreement between parents and children seems to be lower for latent traits that parents are unable to directly observe, such as emotional status and social functioning. Parents of children with chronic conditions score perceived health and HRQOL lower than the children themselves, while the opposite has been seen in relatively healthy populations [3-5]. Thus, there are strong arguments for obtaining information from both parents and children whenever possible [6]. In situations where a child is either unable or unwilling to complete a self-report measure, the use of a parent report may be the only alternative.

A necessary condition for assessing PRO is to develop sound, reliable and valid measures to capture health status from the perspective of parents and children. One such measure is the Child Health and Illness Profile (CHIP)-Child Edition(CHIP-CE) [7,8], an instrument that collects self-reported and parent-reported health information about children aged 6 to 11. The adolescent version of the CHIP (CHIP-Adolescent Edition, CHIP-AE) [9], which is based on the same conceptual framework as the child version, has been translated into Spanish, culturally adapted, and validated [10,11]. The CHIP-CE has also been translated and adapted in Spain [12] following the international guidelines for cross-cultural adaptations [13].

The aims of the present study were to assess the reliability, and content and construct validity of the Spanish version of the CHIP-CE Parent Report Form (CHIP-CE/PRF), to analyze parent-child agreement, and to compare the results with the original U.S. version. Another manuscript presents the reliability and validity of the Spanish CHIP-CE Child Report Form (CHIP-CE/CRF) (Estrada MD, Rajmil L, Herdman M, Serra-Sutton V, Tebé C, Alonso J, Riley AW, Forrest CB, Starfield B: Reliability and Validity of the Spanish version of the Child Health and Illness Profile (CHIP) Child-Edition, Child Report Form (CHIP-CE/CRF), submitted).

## Methods

### Sample selection and procedures

Parents of all children (6-12 years old) selected to form a representative sample of primary school children from the city of Barcelona during the academic year 2002 to 2003 were invited to participate in the validation study of the CHIP-CE/PRF. A probabilistic sampling selection was conducted following a 2-stage process, in which the primary sample units were schools. Schools were stratified by the type of school (public or private) and by the Family Economic Capacity Index (FECI) of neighborhoods in Barcelona (low, middle and high, grouped in tertiles) [14] which assesses the socioeconomic level of the school, according to the neighborhood in which it is located. In the second stage, classrooms were randomly selected, and all students from each classroom were enrolled in the study. All the primary education grades (1st to 6th year) were included in each stratum. A theoretical sample size of 1300 children and their parents was estimated based on previous experience in the development of the adolescent version and our attempts to reproduce the methods used by the original authors as closely as possible. Non-response was expected to be approximately 20%.

A convenience subsample of 308 parents from two schools (from high and middle socio-economic level, respectively) was selected to administer the Spanish parent version twice, one week apart, and to assess the known group validity.

Parents, preferably mothers, of the students received a letter inviting them to participate in the study together with their son/daughter. Parents filled in the questionnaire at home (average time to complete the Spanish CHIP-CE/PRF was 20 min) and questionnaires were collected at school in sealed envelopes one week later.

All procedures were carried out following the data protection requirements of the European Parliament (Directive 95/46/EC of the European Parliament and of the Council of 24 October 1995 on the protection of individuals with regard to the processing of personal data and on the free movement of such data). The ethical and legal requirements were adhered to, and signed informed consent was requested from the schools and parents of each participating child.

### The parent version of the CHIP

The CHIP is based on a broadly defined conceptual framework which recognizes that health includes not only perceptions of well-being, illness and health but also participation in developmentally appropriate tasks and activities, and behaviors that promote or threaten health. The Spanish version of the CHIP-CE/PRF measures the perceived health of children 6 to 12 years old and comprises 75 items included in 5 domains and 12 sub-domains: Satisfaction domain assesses the overall perceptions of well-being and self-concept (satisfaction with health, 7 items; self-esteem, 4), Comfort includes parents' assessment of the child's experience of physical and emotional symptoms and positive health sensations and observed limitation of activities (physical comfort, 9; emotional comfort, 9; restricted activity, 4), Resilience includes parents' assessment of family support, child's coping abilities, and child's physical activity levels (family involvement, 8; social problem-solving, 5; physical activity, 6), Risk avoidance assesses the degree to which the child does not engage in behaviors that increase the likelihood of future illness or injury or that interfere with social development (individual risk avoidance, 4; threats to achievement, 10) and Achievement includes parents' assessment of the extent to which the child meets expectations for role performance in school and with peers (academic performance, 4; peer relations, 5). The domains and subdomains are scored in the positive meaning of health; that is, higher scores indicate greater satisfaction, comfort, and resilience, less risk, and better achievement.

To facilitate interpretation of the scores and enable comparison of different subgroups of children, the domains and subdomains are standardized to an arbitrary mean of 50 and a standard deviation (SD) of 10. The individual mean of each domain (range, 1-5) is taken into account in the standardization procedure, as well as the group mean and SD in the Spanish version. For example: Satisfaction = (([individual score in Satisfaction - group mean in Satisfaction]/SD of the group) * 10) + 50. The Spanish version of the CHIP-CE/PRF was developed in parallel to the child version, following international guidelines for cross-cultural adaptations [13]. As most of the items come from the adolescent version (CHIP-AE), which was previously adapted in Spain [10], only minor rewording and revision for proxy administration were needed. No cognitive interviews or pilot tests were carried out, since it was assumed that if children and teenagers were able to understand the instrument, parents would also understand it. The only item excluded from the original U.S. version was a question collecting information on homework because this is not a common activity in most Spanish primary schools. Therefore, the Spanish CHIP-CE/PRF includes 75 items instead of the 76 in the original U.S. version. A short format of the Spanish CHIP-CE/PRF containing 44 items in parallel with the child version is also available, although only the results from the 75-item format are presented in this study.

The Spanish parent version of the Achenbach Child Behavioural Checklist (CBCL) was administered to assess emotional and behavioral problems in children [15,16]. CBCL is a standardized instrument for the assessment of child behavior problems. It evaluates clinical subscales of anxiety/depression, social problems, somatic symptoms, isolation, thinking problems, attention problems, criminal conduct, aggressive behavior, and other problems. It also provides a Total Problems score. Criterion validity of the Spanish version was assessed and found to be acceptable against a structured psychiatric interview (area under the receiver operating characteristic = 0.767; IC95%: 0.696 a 0.837). Internal consistency, and test-retest and inter-rater reliability were also acceptable [17]. The CBCL Total Problems score was divided into 2 categories for the purposes of the study: mentally healthy (≤64) and borderline-probable clinical case (>64), using the recommended cut-off points [18].

Information on the characteristics of the schools was collected, and the child's age and gender, and the highest family level of education (primary school, secondary school, or university degree) were collected from parents.

### Statistical analysis

The percentage of missing values and the ceiling and floor effects were determined. Floor and ceiling effects for all domains were assessed by calculating the percentage of respondents scoring the minimum and maximum possible scores on each scale using raw (untransformed) data. Cronbach's alpha coefficient was used to assess internal consistency [19] and the intraclass correlation coefficient (ICC) to analyze test-retest reliability [20]. The ceiling and floor effects were expected to be no more than 15%, and a minimum of 0.70 was set as an acceptable reliability criterion for internal consistency [21] and the test-retest ICC [22].

Construct validity was examined by determining whether parents perceived their child's health in the predicted directions according to a priori hypotheses. According to the literature review and previous hypotheses with the original version [7,8], it was expected that younger children would score higher in Satisfaction than older children, that girls would have lower (worse) scores in Comfort and higher (better) scores in Risk Avoidance than boys, and that children with a disadvantaged socioeconomic status would have lower (worse) scores in Comfort and Resilience than their peers with an advantaged socioeconomic status. Scores for the Spanish CHIP-CE/PRF domains and 95% confidence intervals (95% CI) were computed by age groups (6-7 years, 8-12 years), gender, and socioeconomic status, based on the highest level of education attainment of either parent. Standardized mean score differences in the Spanish CHIP-CE/PRF domain and subdomain scores were analyzed using the effect size (ES) [23], classified as no effect (<0.2), and low (0.2-0.5), moderate (0.51-0.8) or high effect (>0.8).

Known group validity was analyzed by comparing the standardized mean scores and 95% CIs between children whose parents scored within the normal range on the CBCL and their counterparts in the borderline-clinical range. Standardized mean score differences in the Spanish CHIP-CE/PRF domains were analyzed using the ES [21]. Based on the general similarity of content between the CHIP Comfort domain and the scales in the CBCL, we expected to see the highest ES between healthy and borderline probable clinical cases on the Comfort domain. However, we also expected to see some differences, though likely smaller differences, between these two groups on the other CHIP domains because they also measure aspects which could be relevant in discriminating between groups with and without mental health problems. For example, the CHIP Risk Avoidance domain covers several aspects related to conductual problems which could also be reflected by the CBCL.

Parent-child agreement on the Spanish CHIP-CE/PRF was assessed using ICC values. This analysis was conducted for the whole sample and stratifying by two age groups (6-7 years, 8-12 years). Higher CCI was expected in younger children and in the domains assessing more observable aspects (Risk Avoidance and Resilience).

In our study, the primary sampling unit was the school (classified into two strata), and the second unit was the classroom. In order to take into account the hierarchical sample structure and clustered data, analysis were performed using the Module SPSS Complex Samples.

## Results

The overall response rate was 67% (871 participants from 1307 initially selected children and parents), and 61% and 67% for the subsample used to analyze construct validity (n = 188) and test-retest reliability (n = 228, from a total of n = 308). Five children older than 12 years and 1 parent questionnaire without the child response were excluded from further analysis. The response rate was higher in older children and in families from more affluent school areas. The mother was the responding parent in 88% of cases and the mean age of the respondent was 40.2 y (4.9 SD); 52% of children were girls, and 75% were children 8 to 12 years old; a university degree was the highest family level of education in 44% of the sample. The subsample used to analyze construct validity and test-test reliability had a higher parental level of education compared to the whole sample (Table 1).

**Table 1:** Characteristics of the overall sample and subsamples selected to assess construct validity and test-retest

	Total	Construct validity	Test-retest
Total, n	865	188	228
Parents' age, mean (standard deviation)	40.3(4.9)	41.4 (3.6)	41.2 (3.5)
Proxy relationship children respondents, %			
Mother (biological or adoptive)	87.9	86.6	86.9
Father (biological or adoptive)	11.4	12.5	12.3
Others (grandmother, stepmother and others)	0.7	0.9	0.8
Children's age (years), %			
6-7	24.7	25.5	25.4
8-12	75.3	74.5	74.6
Children's gender, %			
Sons	48.2	48.3	48.1
Daughters	51.8	51.7	51.9
Highest family level of education, %			
Primary school	17.8	4.0	3.9
Secondary school	38.2	25.3	25.7
University degree	44.0	70.7	70.4
Type of school, %			
Public	35.6	-	-
Private	64.7	100	100
Family economic capacity index, %			
Low (<92.5)	30.8	-	-
Middle (92.5-114)	45.8	44.8	44.6
High (>114)	23.5	55.2	55.4

The internal consistency reliability of the Spanish CHIP-CE/PRF and the results of the original U.S. version are shown in Table 2. No floor effect was observed. The ceiling effect was higher than 15% in the subdomains of self-esteem (17.8%), restricted activities (70.3%), and individual risk avoidance (25.0%). Internal consistency reliability ranged from 0.68 in the Resilience domain to 0.84 in the Comfort domain. Cronbach alpha coefficients were below the cut-off of 0.7 in 4 subdomains (physical comfort, physical activity, individual risk avoidance, and peer relations). In general, internal consistency was slightly lower than in the original U.S. version. ICCs of the domains ranged from 0.63 (Comfort) to 0.85 (Achievement) and were below 0.7 in 4 subdomains (physical comfort, restricted activity, social problem-solving, and individual risk avoidance), ranging from 0.46 to 0.85. These figures were also slightly lower than the U.S. results (Table 3).

**Table 2:** Missing values, floor and ceiling effects, internal consistency coefficients of the Spanish version of the CHIP-CE/PRF, and results of the original U.S. version*

Domain Subdomain (no. of items)	Spanish version CHIP-CE/PRF (n = 865)						U.S. version* CHIP-CE/PRF (n = 583)
	Missing values	Floor effect (%)	Ceiling effect (%)	Cronbach's alpha coefficient			
				
				6-7 y	8-12 y	Total	Total
**Satisfaction **(11 items)	0	0	2.8	0.79	0.76	0.77	0.84
Satisfaction with health (7)	0	0	6.0	0.73	0.70	0.71	0.74
Self-esteem (4)	0.8	0.1	17.8	0.75	0.68	0.70	0.86
**Comfort **(22)	0	0	1.4	0.83	0.85	0.84	0.88
Physical comfort (9)	0	0	9.8	0.64	0.70	0.69	0.76
Emotional comfort (9)	0	0	5.7	0.82	0.83	0.82	0.85
Restricted activity (4)	0.1	0	70.3	0.85	0.87	0.87	0.88
**Resilience **(19)	0	0	0	0.71	0.68	0.68	0.79
Family involvement (8)	0	0	1.6	0.69	0.71	0.70	0.75
Social problem-solving (5)	0.7	0.3	5.9	0.78	0.71	0.73	0.81
Physical activity (6)	0	0	3.4	0.53	0.59	0.58	0.71
**Risk Avoidance **(14)	0	0	1.5	0.81	0.77	0.78	0.82
Individual risk avoidance (4)	0.1	0	25.0	0.61	0.48	0.53	0.68
Threats to achievement (10)	0	0	2.7	0.79	0.76	0.77	0.80
**Achievement **(9)	0	0	1.0	0.72	0.76	0.75	0.83
Academic performance (4)	0.3	0.1	15.0	0.87	0.86	0.86	0.86
Peer relations (5)	0.1	0	3.9	0.57	0.65	0.63	0.75

*See reference 8

**Table 3:** Test-retest reliability of the Spanish version of the CHIP-CE/PRF and results from the original U.S. version*

CHIP-CE/PRF Domain Subdomain	Intraclass Correlation Coefficient			
	Spanish version n = 228			U.S. version* (n = 190)
	
	Total	6-7 y	8-12 y	Total
**Satisfaction **	0.76	0.75	0.76	0.79
Satisfaction with health	0.69	0.71	0.70	0.78
Self-esteem	0.72	0.74	0.71	0.71
**Comfort **	0.63	0.63	0.60	0.71
Physical comfort	0.59	0.59	0.62	0.63
Emotional comfort	0.68	0.75	0.66	0.74
Restricted activity	0.46	0.45	0.47	0.36
**Resilience**	0.77	0.83	0.76	0.80
Family involvement	0.76	0.83	0.72	0.78
Social problem-solving	0.54	0.69	0.45	0.74
Physical activity	0.71	0.73	0.70	0.75
**Risk Avoidance **	0.69	0.75	0.68	0.84
Individual risk avoidance	0.63	0.66	0.60	0.70
Threats to achievement	0.70	0.78	0.66	0.82
**Achievement **	0.85	0.84	0.85	0.85
Academic performance	0.85	0.87	0.85	0.77
Peer relations	0.74	0.78	0.72	0.82

*See reference 8

Younger girls had higher (better) scores in the Academic achievement subdomain (ES = 0.43), and the Satisfaction domain (ES = 0.33) than older girls, the latter at limits of statistical significance. Older girls had higher (better) scores in the Risk Avoidance domain than boys at all ages. Younger boys and girls had higher score in the Family involvement subdomain than their older counterparts. Children from families with a university degree had higher scores in the Achievement domain and Physical comfort and Academic performance subdomains than their counterparts whose families were in the primary school category (ES = 0.36, 0.44 and 0.53, respectively) (Table 4).

**Table 4:** Standardized mean domain and subdomain scores and 95% confidence intervals (95%) of Spanish CHIP-CE/PRF version by gender, age, highest family level of education, and effect size (ES) (n = 865)

CHIP-CE/PEF domains						
***Age and gender ***	**Sons (n = 417)**			**Daughters (n = 448)**		

	**6-7 y** **(n = 111)**	**8-12 y** **(n = 306)**	**ES** **(younger vs. older)**	**6-7 y** **(n = 103)**	**8-12 y** **(n = 345)**	**ES** **(younger vs. older)**

**Satisfaction**	50.9 (48.7-53.0)	50.4 (49.5-51.3)	0.06	52.7 (49.5-55.8)	48.7 (47.3-49.9)	0.33
Satisfaction with health	50.9 (49.7-52.2)	50.4 (49.5-51.4)	0.07	51.4 (48.3-54.6)	49.0 (47.6-50.2)	0.21
Self-esteem	50.7 (48.2-53.2)	50.2 (49.3-51.2)	0.05	52.8 (49.9-55.6)	48.8 (47.5-50.0)	0.35
**Comfort**	50.4 (48.2-52.6)	50.2 (49.1-51.3)	0.02	48.6 (46.1-51.2)	50.1 (48.9-51.4)	0.13
Physical comfort	50.7 (48.7-52.6)	50.9 (49.8-51.9)	0.02	47.7 (45.3-50.1)	49.7 (48.7-50.8)	0.21
Emotional comfort	50.1 (47.5-52.7)	49.7 (48.4-51.0)	0.03	50.6 (48.2-52.9)	50.0 (48.8-51.3)	0.05
Restricted activity	50.2 (48.1-52.2)	49.9 (48.9-51.0)	0.03	48.3 (46.0-50.6)	50.5 (49.4-51.6)	0.23
**Resilience**	49.6 (47.7-51.5)	50.3 (49.4-51.3)	0.10	51.5 (47.4-55.5)	49.4 (48.1-50.7)	0.16
Family involvement	53.6 (52.6-54.7)	49.3 (48.2-50.5)	0.49	53.3 (50.2-56.4)	48.4 (47.2-49.6)	0.43
Social problem-solving	47.2 (45.5-48.9)	48.7 (47.5-49.8)	0.16	52.1 (48.4-55.7)	51.4 (50.1-52.8)	0.05
Physical activity	50.0 (47.9-52.1)	53.3 (51.9-54.8)	0.30	46.9 (45.6-48.2)	48.0 (46.9-49.0)	0.12
**Risk Avoidance**	45.8 (43.0-48.7)	48.1 (46.6-49.7)	0.18	52.0 (48.4-55.6)	52.4 (51.2-53.5)	0.03
Individual risk avoidance	45.1 (42.3-48.0)	49.5 (48.1-50.9)	0.37	49.7 (46.3-53.2)	52.1 (50.8-53.4)	0.19
Threats to achievement	48.1 (45.8-50.3)	47.3 (45.8-48.9)	0.06	53.8 (50.8-56.7)	51.8 (50.9-52.8)	0.19
**Achievement**	50.1(48.2-52.1)	49.0 (47.5-50.4)	0.11	53.5 (50.7-56.3)	49.9 (48.5-51.2)	0.31
Academic performance	50.8 (48.3-53.4)	48.8 (47.3-50.3)	0.17	54.0 (51.1-57.0)	49.6 (48.5-50.7)	0.43
Peer relations	48.9 (47.8-49.9)	49.7 (48.7-50.7)	0.11	50.8 (48.7-52.9)	50.4 (48.9-51.9)	0.03

***Highest family level of education***	**Primary school** **(n = 150)**	**Secondary school** **(n = 322)**	**University degree** **(n = 371)**	**ES** **(secondary vs. primary school)**	**ES** **(university vs. secondary school)**	**ES** **(university vs. primary school)**

**Satisfaction**	51.6 (49.8-53.3)	50.7 (49.4-51.9)	48.9 (47.9-49.9)	0.08	0.18	0.28
Satisfaction with health	51.3 (49.7-52.7)	50.9 (49.8-52.3)	48.8 (47.9-49.8)	0.03	0.19	0.27
Self-esteem	51.5 (49.5-53.5)	50.3 (49.2-51.4)	49.2 (48.2-50.2)	0.12	0.12	0.23
**Comfort**	48.1 (46.3-49.9)	50.1 (48.8-51.4)	50.8 (49.8-51.8)	0.18	0.07	0.28
Physical comfort	47.5 (46.2-48.8)	50.3 (49.0-51.5)	51.1 (52.2-52.0)	0.28	0.09	0.44
Emotional comfort	49.7 (47.8-51.6)	50.0 (48.7-51.3)	50.1 (48.8-51.3)	0.02	0.00	0.03
Restricted activity	48.1 (46.4-49.8)	50.0 (48.7-51.3)	50.8 (49.9-51.7)	0.17	0.08	0.31
**Resilience**	49.3 (47.5-51.1)	50.5 (49.5-51.6)	49.9 (49.2-50.7)	0.13	0.07	0.08
Family involvement	49.0 (46.8-51.3)	50.2 (48.8-51.6)	50.2 (49.1-51.4)	0.12	0.00	0.10
Social problem-solving	50.2 (48.7-51.6)	50.3 (49.1-51.4)	49.8 (48.8-50.8)	0.01	0.05	0.04
Physical activity	49.3 (47.5-51.2)	50.6 (49.3-51.8)	50.0 (49.1-51.0)	0.12	0.05	0.08
**Risk Avoidance**	51.4 (49.2-53.7)	49.8 (48.5-51.1)	49.5 (48.1-51.0)	0.14	0.02	0.14
Individual risk avoidance	52.4 (50.7-54.2)	49.8 (48.5-51.2)	49.0 (47.3-50.8)	0.23	0.06	0.24
Threats to achievement	49.8 (47.4-52.3)	49.8 (48.6-51.0)	50.3 (49.0-51.5)	0.00	0.04	0.03
**Achievement**	47.5 (45.2-49.8)	49.3 (48.3-50.4)	51.8 (50.6-52.9)	0.17	0.24	0.36
Academic performance	46.5 (44.1-48.8)	48.8 (47.8-49.8)	52.7 (51.5-53.8)	0.22	0.39	0.53
Peer relations	50.7 (49.0-52.3)	50.6 (49.6-51.7)	49.2 (48.0-50.4)	0.01	0.13	0.13

Mean domain scores are standardized to an arbitrary mean of 50 and 1 SD = 10.

The standardized mean domain scores of the Spanish CHIP-CE/PRF according to the overall CBCL scale scores are shown in Table 5. The highest ES was seen in the Comfort domain (1.45), although lower scores on the CHIP were also found on all of the other domains in borderline/probable clinical cases compared to mentally healthy children.

**Table 5:** Standardized mean domain scores and 95% confidence intervals (95% CI) of the Spanish CHIP-CE/PRF by children's mental health status reported by parents (CBCL Total Problems score)*, and effect sizes (ES) (n = 188)

**CHIP-CE/PRF Domains**			
	**Healthy mental** **(n = 167)**	**Borderline-Probable clinical case (n = 21)**	**ES** **(Healthy mental vs. Borderline clinical)**
	
	**Mean (95% CI)**	**Mean (95% CI)**	
	
Satisfaction	50.3 (48.8 - 51.8)	40.6 (35.6 - 45.5)	0.98
Comfort	54.4 (53.2 - 55.6)	42.7 (37.6 - 47.8)	1.45
Resilience	52.2 (50.6 - 53.7)	47.8 (43.8 - 51.8)	0.45
Risk avoidance	55.3 (54.0 - 56.6)	44.0 (39.9 - 48.2)	1.37
Achievement	54.2 (52.8 - 55.6)	44.6 (40.7 - 48.6)	1.08

CBCL, Achenbach Child Behavioral Checklist

Mean domain scores are standardized to an arbitrary mean of 50 and 1 SD = 10.

*CBCL Total Problems score: ≤64 healthy mental and >64 borderline-clinical probable case

The level of parent-child agreement of the Spanish CHIP-CE/PRF was low for all domains (0.22-0.37). Correlations were slightly higher for all domains in the oldest age group (Table 6).

**Table 6:** Agreement parent-child in the Spanish CHIP-CE (n = 865)

	Intraclass Correlation Coefficient		
CHIP-CE/PRF *Domain*	Total (n = 865)	6-7 y (n = 214)	8-12 y (n = 651)
Satisfaction	0.31	0.24	0.31
Comfort	0.22	0.14	0.26
Resilience	0.25	0.16	0.30
Risk Avoidance	0.32	0.26	0.34
Achievement	0.37	0.33	0.38

## Discussion

The results of this study suggest that the parent version of the Spanish CHIP-CE has acceptable psychometric properties although further research is needed to check reliability at sub-domain level. The CHIP-CE parent report form provides a comprehensive, psychometrically sound measure of health for Spanish children 6 to 12 years old. It can be a complementary perspective to the self-reported measure or an alternative when the child is unable to complete the questionnaire. In general, the results are similar to the original U.S. version. The Spanish CHIP-CE/PRF showed acceptable reliability at domain level and also acceptable content and construct validity.

The Spanish parent version of the CHIP shows acceptable ability to differentiate in the expected direction between groups known to be in better or poorer health according to sociodemographic factors and health characteristics (age, gender, socioeconomic status, and mental health), with some exceptions. For example, the hypotheses regarding differences in Risk Avoidance and Resilience according to the family level of education were not confirmed. This could be partly related to response bias if the non-responses, which were more frequent in the low socioeconomic group, were associated with poor health status. On the other hand, some authors have found fewer socioeconomic differences in health at this age period than later in adolescence [24,25]. Of note, although the subsample analyzed was small, the highest ES was observed in children with a probable mental health problem compared to their healthy counterparts in the Comfort domain of the CHIP and the differences were even greater than those seen in the child version (Estrada MD, Rajmil L, Herdman M, Serra-Sutton V, Tebé C, Alonso J, Riley AW, Forrest CB, Starfield B: Reliability and Validity of the Spanish version of the Child Health and Illness Profile (CHIP) Child-Edition, Child Report Form (CHIP-CE/CRF), submitted). In this sense, moderate associations found between Total Problems (CBCL) and other domains of the CHIP would be expected given the negative impact of mental health problems on daily functioning, although these measures represent different concepts. These findings suggest that both the parent and the child version can be useful in studies analyzing mental health in children.

There are some differences between this study examining the Spanish version and the one validating the U.S. version. The most important include the fact that the Spanish sample was a representative urban group whereas the U.S. sample came from different settings, and the slightly different analytical strategy used: the effects size instead of correlation coefficients. Although the internal consistency coefficients of the Spanish version were acceptable, they were slightly lower in some subdomains than the U.S. version, specifically in the Resilience domain. The specific subdomains below the standard recommendations were similar in both versions. Resilience is a complex construct that includes individual, family and community factors, with some similarities and many differences regarding the concept of HRQOL [6]. It is a concept difficult to capture in a single score because it refers to the child's disposition and behavior that is likely to enhance future health [26]. In the US version, the results for this domain were also suboptimal. Nonetheless, the Spanish Resilience domain presented acceptable test-retest stability.

The CHIP has several advantages given that it was developed following a broad conceptual framework. The instrument was designed to combine several c*o*ncepts and constructs such as illness/health status, HRQOL, resilience and achievements in one single instrument, based on explicit theory and supported by a substantial empirical findings [27].

Strengths of the study include the fact that the psychometric properties of the Spanish version of the instrument were assessed in a large representative sample of urban primary school children and their parents, including a wide range of socioeconomic status with low, middle and high income families all substantially represented, and families from both public and private schools. Furthermore, this study has made available in Spain one of very few instruments that can be used in younger age groups, for example those in the 6-7 year range. The fact that the sample was large also meant it was possible to analyze parent-child agreement specifically in this younger age group. Parent-child agreement in such young age groups has not been widely studied.

The availability of the Spanish parent version of the CHIP-CE allows assessment from a multi-informant perspective as a complement to the self-reported version, without substituting it. The present study also reinforces the use of both versions in parallel, mainly in specific situations. For example, children with certain conditions, such as attention deficit hyperactivity disorder (ADHD), might be less aware of their health problems. A longitudinal study using child self-rating and parent reporting in children with ADHD [28] showed that the children scored close to the general population values, whereas their parents scored more than one SD below the general population mean on most of the Spanish CHIP-CE domains and subdomains. After their children had received 8 weeks of treatment, however, parents scored close to the population mean. This study provided a more complete clinical picture than if information had been collected from only one perspective on perceived health. The figures from these studies, and another study using a different child health instrument [29] showed low parent-child agreement in all domains of health, A recent literature review on HRQOL instruments in children [30] found 13 generic instruments with self- and parent-reported versions, and only 6 of which demonstrated acceptable psychometric properties. Availability of both a self-reported and parent-reported Spanish CHIP-CE would be an opportunity to analyze inconsistencies between child and parent reports more in depth.

The results of the present study can also be useful in future studies. Interpretation of the CHIP-CE scores can be facilitated by comparing the values from our reference population sample with that of other specific population subgroups. In addition, the instrument can be used to develop a health classification system that will broaden its application. One advantage of the health-profile types developed with the original U.S. version [31,32] and with the Spanish adolescent version of the CHIP [33] is that they enable easy capture of the multidimensional nature of health. The Spanish child version will incorporate this age group in the development of health profile types in the near future.

The study had some limitations. Validity and reliability have been assessed in a large, heterogeneous, urban sample, but further research is needed to compare the domain and sub-domain scores of the CHIP in children and parents from other settings. Secondly, although school sampling represents a frequently used, efficient and less time consuming method to collect representative samples of school-age children, cluster sampling usually results in a lack of independence of observations obtained from units within the same cluster [34]. Consequently, in order to obtain valid estimates of variability, analyses should account for these correlated data as well as the multistage sampling design. In this study, data analysis accounted for the complex survey design, thereby yielding parameter and variability estimates that would allow for valid inferences about the population that was sampled. Moreover, these analyses can be considered as a conservative procedure given that increases the standard error. Thirdly, the sub-sample used to assess known groups' validity and test-retest reliability had a relatively small proportion of families in the lower levels of education, which may have affected results on these two properties. Finally, the fact that few health status instruments for younger children have been adapted and validated in Spain limited the possibility of a more in-depth assessment of construct and convergent validity, mainly in 6-7 year old category where at the time the study was performed no instruments had been adapted for use in Spain.

The Spanish version of the CHIP-CE/PRF shows promise as a useful instrument for assessing health status from childhood through adolescence in parallel with the child version and together with the adolescent version. Future studies should analyze the criterion validity and sensitivity to change of the Spanish CHIP-CE/PRF, and investigate its application in the clinical setting. Longitudinal studies would help to determine its value in the predictive assessment of future health. Future research should also focus on parent-child agreement using a modern test theory, such as differential item functioning (DIF), to avoid bias due to specific subgroup characteristics and confirm the differences found in previous studies [7].

In conclusion, the Spanish version of the CHIP-CE/PRF has shown acceptable coefficients of reliability and validity that are similar to those of the original U.S. version. Although the reliability of some sub-domain scores requires further investigation, the Spanish CHIP-CE/PRF shows promise as a measure of health status, and will be particularly useful in providing information on the evolution of health status from childhood through adolescence, when used in conjunction with the adolescent version.

## Competing interests

The authors declare that they have no competing interests.

## Authors' contributions

MDE, LR, VS, CT and JA participated in the conception and design of the study. MDE, LR, JA, VS, and CT analyzed the data. MDE, LR, VS, MH, JA, AR, CF, BS and MH participated in the drafting of the article. All authors contributed to a critical revision of the manuscript and made a substantial contribution to its content, and all authors read and approved the final manuscript.
